# *De novo* transcriptome analysis of somatic embryogenesis induced by plant hormones combined with adenosine 5-monophosphate and nicotinamide adenine dinucleotide molecules in *Aspilia africana* (Pers.) C.D.Adams

**DOI:** 10.1016/j.jgeb.2026.100687

**Published:** 2026-03-27

**Authors:** Roggers Gang, Ariranur Haniffadli, Daeui Park, Chunggeon Lee, Subramanian Muthamil, Jun Hong Park, Yeongjun Ban, Youngmin Kang

**Affiliations:** a Korean Convergence Medical Science Major, University of Science and Technology (UST), Daejeon 34113, South Korea; b Herbal Medicine Resources Research Center, Korea Institute of Oriental Medicine (KIOM), Geonjae-Ro, Naju-Si 58245, South Korea; c National Agricultural Research Organization (NARO), National Semi-Arid Resources Research Institute (NaSARRI), Soroti, Uganda; d Center for Biomimetic Research, Division of Advanced Predictive Research, Korea Institute of Toxicology, Daejeon 34114, South Korea; e Department of Physiology, College of Veterinary Medicine, Jeonbuk National University, Iksan 54596, South Korea

**Keywords:** *Aspilia africana*, Comparative transcriptome, *De novo* assembly, Differentially expressed genes, RNA-seq, Somatic embryogenesis

## Abstract

**Background:**

*Aspilia africana* (Pers.) C.D.Adams is an important medicinal plant in Africa. In a previous study, we induced direct somatic embryogenesis (SE) from its leaf explants, by combining plant hormones with adenosine 5′-monophosphate (AMP) and nicotinamide adenine dinucleotide (NAD). SE is a valuable biotechnological tool with various applications such as synthetic seed production, and understanding molecular events that induce SE is vital for its effective manipulation and improvement. Therefore, this study aimed to elucidate the molecular basis of SE induced by a combination of plant hormones and other mitogenic molecules (AMP and NAD) in *A. africana*. Using NovaSeq 6000 (Illumina, Inc., USA) technology, we conducted *denovo* transcriptome sequencing of leaf tissues representing three distinct SE developmental stages: non-embryonic leaf (NEL), globular embryo formed leaf (GEL), and cotyledonary embryo formed leaf (CEL).

**Results:**

A total of 8669 genes were differentially expressed in the three samples, and transcripts encoding plant hormone signalling components including auxin and cytokinin were markedly enriched. SE candidate genes such as SERK2, ERF12, ERF2, and LEA14-A were up-regulated in embryogenic tissues. Additionally, qRT-PCR analysis confirmed the expression levels of some selected SE related genes.

**Conclusions:**

For the first time, this study reports a *de novo* transcriptome assembly and gene expression analysis during SE induction in *A. africana*. The work also provides insights into SE induced by a combination of plant hormones with other mitogenic molecules, and findings suggest that the mechanism of SE induction in this case may be the same or similar to that induced by only plant hormones. Overall, this study offers beneficial resources for future molecular research on *A. africana*, and may be a key resource platform for future functional research in plant embryogenesis.

## Highlights

• First molecular study of *Aspilia africana* by *denovo* transcriptome sequencing.
• Insights into somatic embryogenesis induced by plant hormones combined with other mitogenic molecules.
• Somatic embryogenesis candidate genes including SERK2, ERF12, and LEA14-A were up-regulated.
• Observed similar mechanisms to that of somatic embryogenesis induced by plant hormones alone.

## Graphical abstract

**Figure:**
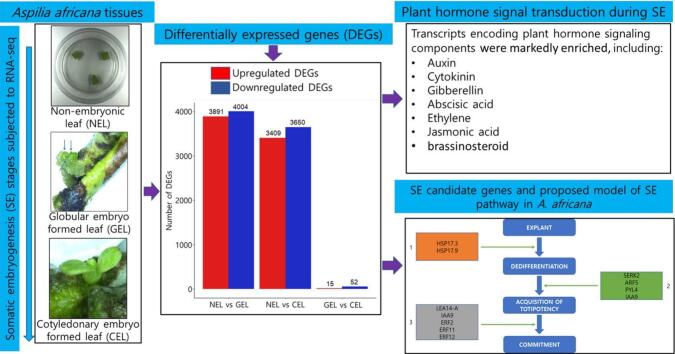

## 1 Introduction

*Aspilia africana* (Pers.) C. D. Adams, a shrub within Asteraceae family has been used in many African communities to traditionally treat various disease conditions over the years ^1^. These include wounds, diabetes mellitus, gonorrhea, malaria, stomach ache, measles, osteoporosis, tuberculosis, and gastric ulcers ^2^. Indeed, pharmacological evaluations show that *A. africana* possess a range of bioactivities such as antimalarial, antimicrobial, antidiabetic, wound-healing, anti-inflammatory, anticancer, and anti-ulcer effects 3, 4. Beyond its medicinal value, *A. africana* also serve as an important source of forage for livestock including cattle, chicken, and goats, and it is regarded as one of the plants most commonly grazed by these animals in Africa ^1^. However, the growing demand for both therapeutic and forage uses, driven by rapid human population growth, is leading to a decline in the wild populations of *A. africana*, highlighting the need for its propagation and conservation 3, 4. Unfortunately, propagation of *A. africana* from seed is challenging due to low rate of germination in different types of soil 3, 4.

As an alternative propagation strategy, we previously established a regeneration method of *A. africana* via direct somatic embryogenesis (SE) from leaf explants ^5^. SE is the process where somatic cells dedifferentiate to form somatic embryo cells and subsequently the organism ^6^. Direct SE involves induction of somatic embryos without progression through a callus phase, whereas indirect SE involves the formation of somatic embryos via an intervening callus stage ^7^. SE is influenced by a number of factors, and it is induced by subjecting explants to exogenous growth regulators or stress conditions ^8^. Although plant hormones are recognized as the primary drivers of SE induction, supplementation of culture media with other exogenous molecules like adenosine 5′-monophosphate (AMP) and nicotinamide adenine dinucleotide (NAD) has been used to enhance SE in several plant species ^9^. These exogenous molecules not only regulate plant growth similar to phytohormones but also contribute to processes like reproduction, stress responses, nucleic acid synthesis, and general cellular metabolism ^10^. Adenosine phosphate-isopentenyl transferase uses AMP, ADP, or ATP together with dimethylallyl diphosphate or hydroxymethyl-butenyl diphosphate to catalyze the first step in isoprenoid cytokinin biosynthesis ^11^. NAD is an important cofactor that participates in numerous key cellular functions, and it is the major source of nicotinamide in plant cells 12, 13. Both AMP and NAD have been reported to improve the SE of some recalcitrant plant species, including *Peucedanum japonicum* Thunberg and *Rosa hybrids* L., respectively 9, 14.

SE is a valuable biotechnological tool used in various applications such as synthetic seed production, clonal plant regeneration, cryopreservation, and genetic improvement ^6^. The comparable developmental pattern of zygotic and somatic embryos make SE a suitable system for examining zygotic embryogenesis at various biological levels ^8^. Although SE is highly valuable, the phenomenon is still not well understood because of unclear mechanisms at cellular level, necessitating further SE molecular studies for improved outcome 15, 16. In line with *A. africana* SE, key limitations include asynchronous embryo formation and a low rate of conversion from somatic embryos to viable plantlets ^5^. In this case, molecular studies including transcriptomics and genomics may be essential for addressing these challenges by helping identify molecular markers that can be used to enhance *A. africana* SE through targeted manipulation ^5^.

Different molecular biology technologies such as microarray-based methods, gene cloning, and suppression subtractive hybridization, have been employed to examine gene activities during SE, enabling identification of SE associated genes, for example, Leafy Cotyledon (*LEC1* and *LEC2*) and Somatic Embryogenesis Receptor Kinases (*SERKs*) in cotton ^17^, Valencia sweet orange ^18^, and Arabidopsis ^19^. In recent decades, development of next generation sequencing approaches, like RNA sequencing (RNA-seq), provide more comprehensive and less biased insights into actively expressed genes within the transcriptome compared to previous methods ^20^. RNA-seq is most commonly used to analyze differential gene expression and it has been applied in many plants’ SE studies at molecular level 8, 21, 22. Notably, regulation of genes during hormone-induced SE has been the predominant focus of most of these studies.

To the best of our knowledge, transcriptome profiling of SE in *A. africana* has not been previously reported. Furthermore, the molecular effects of combining plant hormones with other mitogenic molecules, such as AMP and NAD in embryogenic cultures have not been thoroughly characterized in plants. Therefore, this study aimed to elucidate the molecular basis of SE in *A. africana* by examining transcriptome profiles during direct SE induced by a combination of plant hormones and mitogenic molecules (AMP and NAD). Using NovaSeq 6000 (Illumina, Inc., USA) technology, we separately conducted *denovo* transcriptome sequencing of leaf tissues from three distinct SE developmental stages of non-embryonic leaf (NEL) at day 0 of culture, globular embryo formed leaf (GEL) at 21 days of culture, and cotyledonary embryo formed leaf (CEL) at 28 days of culture. Of note is that *de novo* transcriptome sequencing was chosen in this study because the species (*A. africana*) lacks genome resources and a thorough assessment of its global transcription. For the first time, this study reports a *de novo* transcriptome assembly and gene expression analysis during SE induction in *A. africana*, and provides insights into SE induced by a combination of plant hormones and other molecules (AMP and NAD). Taken together, this work offers beneficial resources for future genomic, genetic, and transcriptomic research on *A. africana*, and could prove essential for future functional investigations on plant embryogenesis.

## 2 Method and materials

### 2.1 Plant material and initiation of tissue culture

Direct SE in *A. africana* was induced following our previously established method ^5^. Leaves of *A. africana* (used as explants) were obtained from two-month-old *in vitro* plants (derived from seeds) at Korea Institute of Oriental Medicine (KIOM), Herbal Medicine Resources Research Center, Naju, Republic of Korea. To induce SE, leaf explants (about 1 cm^2^) were cultured (adaxial surface facing the medium in crystal-grade polystyrene Petri dishes (100 × 20 mm)) for twenty-four days on Murashige and Skoog (MS) medium fortified with 1.0 mg/L benzylaminopurine (BAP), 3.472 x 10^-2^ mg/L AMP, and 3 g/L gelrite. To differentiate and mature the somatic embryos, leaf explants with embryos from induction medium (after 24 days of culture) were transferred to MS medium supplemented with 0.5 mg/L abscisic acid (ABA), 6.634 x 10^-2^ mg/L NAD, and 9 g/L gelrite, for ten days. Both media (for induction and maturation) contained 30 g/L sucrose and pH was made 5.7–5.8 prior to autoclaving for 20 min at 121 °C. Each container had three leaf explants and incubation of all cultures occurred at 16 h photoperiod and 80% relative humidity. Sample leaf tissues (two replicates per treatment) were collected at three developmental stages during SE in *A. africana* at day 0-NEL, day 21-GEL, and day 28-CEL. Accordingly, leaf tissues sampled at 21 days (GEL) and 28 days (CEL) had developed somatic embryos, thus GEL and CEL samples represent mixed leaf and embryo tissues without separation, while NEL samples comprised only leaf tissues (At day 0, the leaf tissues had not developed somatic embryos, and this served as control). After collection, the samples were promptly frozen and kept at −80 °C for transcriptome analysis.

### 2.2 RNA isolation

Total RNA was isolated using Trizol reagent (Invitrogen). TapeStation 4000 System (Agilent Technologies, Amstelveen, The Netherlands) and ND-2000 Spectrophotometer (Thermo Inc., DE, USA) were used to evaluate the quality and quantity of RNA, respectively.

### 2.3 Preparation of library and sequencing

Using the NEBNext Ultra II Directional kit (NEW ENGLAND BioLabs, Inc., UK), total RNA was used to construct libraries. mRNA isolation was performed with the Poly(A) RNA Selection Kit (LEXOGEN, Inc., Austria), and the isolated mRNAs were utilized for cDNA synthesis and shearing, as per the manufacture’s guideline. Indexing was carried out with Illumina indexes 1–12, and the enrichment step was performed by PCR. Next, libraries were examined with the TapeStation HS D1000 ScreenTape (Agilent Technologies, Amstelveen, The Netherlands) to assess the mean fragment size. The library quantification kit was used for quantification on a StepOne Real-Time PCR System (Life Technologies, Inc., USA). Using NovaSeq 6000 (Illumina, Inc., USA), high-throughput sequencing was conducted as paired-end 100 sequencing. All raw sequencing reads have been submitted to the NCBI Sequence Read Archive under accession number PRJNA1334589.

### 2.4 Transcriptome assembly and gene annotation

To remove foreign contaminants present in the sequences, kraken2 ^23^ was used with Standard database. Foreign contamination was removed from the sequences, and adapter and low-quality sequences were filtered out using Fastp ^24^ to generate clean reads. Using default settings, De novo transcriptome assembly was performed with Trinity (v. 2.15.1) ^25^. TransDecoder (v5.7.0) was used to predict coding regions of the assembled transcripts, employing the −-single_best_only parameter. The CD-HIT program (v. 4.8.1) ^26^ with 0.99 sequence identity threshold option was used to reduce transcript redundancy. BLAST (E-value = 1e-5) was used to functionally annotate assembled transcripts against the Swiss-Prot database. Subsequently, the InterProScan ^27^ was performed to assign gene ontology (GO) terms of assembled transcripts.

Similarly, using OmicsBox (https://www.biobam.com/omicsbox/), the BLAST program (E-value = 1e-5) was performed between assembled transcripts and NCBI Nr (non-redundant) database. GO terms were assigned via Blast2GO (E-value = 1e-5) using Nr annotation as reference. The BLAST results in Nr database were further analyzed for E-value distribution, similarity distribution, as well as best-hit species distribution. Biological pathways and processes were examined using Kyoto Encyclopedia of Genes and Genomes (KEGG) ^28^.

### 2.5 Data analysis

Clean reads (obtained from two replicates per sample group-NEL, GEL, and CEL) used for RNA de novo assembly were used for quantification using Salmon ^29^. Using the Python “conorm” package 1.2.0, read counts were normalized by the TMM + CPM method ^30^, and data extraction and graphical display were conducted using ExDEGA (Ebiogen Inc., Korea).

Additionally, differential gene expression between non-embryogenic and embryogenic leaf tissues were estimated using edgeR (v4.0.16), Bioconductor package. To determine the threshold P-value which corresponds to a differential gene expression test, False Discovery Rate (FDR) was applied. Genes with p ≤ 0.001, FDR ≤ 0.01, |fold changes| ≥ 2, and Log_2_(Counts Per Million (CPM)) > 0 were considered to be differentially expressed. Jvenn package was used to generate the venn diagram for DEGs. Furthermore, metascape tool (http:// metas cape. org) as per Zhou et al ^31^ was used to conduct the GO and KEGG pathway enrichment analysis of DEGs. The metabolic pathways of the DEGs were investigated using the KEGG database (http://www.genome.jp/kegg/).

### 2.6 Quantitative Real-Time PCR (qRT-PCR) analysis

qRT-PCR analysis was conducted for 8 genes in order to verify expression of genes observed from RNA-seq. Primer3 Input (https://primer3.ut.ee/) software was used to design specific gene primers (Supplementary material 1) that were commercially synthesized (BIONICS, Seoul, South Korea). Using Prime-Script™ II 1st strand cDNA synthesis Kit (TaKaRa, Dalian, China), total RNA (3 μg) was reversed-transcribed into single-stranded cDNA from each sample. SYBR Green was used to quantify the gene expression and PCR mixture comprised TOPreal qPCR 2 × PreMIX (SYBR Green with low ROX) (TaKaRa, Dalian, China), template DNA, and primers (Supplementary material 1). Actin 7 served as reference standard to calculate and normalize relative quantification of gene expression. qRT-PCR was carried out with CFX96 Touch Real-Time PCR Detection System (Bio-Rad, Hercules, CA, USA). PCR reactions were conducted under the following conditions: initial denaturation for 10 min at 95 °C, denaturation for 10 s at 95 °C, annealing for 15 s at 60 °C, elongation for 15 s at 72 °C, followed by 40 cycles of 95 °C for 15 s and 60 °C for 1 min. To examine the purity of amplified products for each sample, a melting curve was generated. qRT-PCR analysis was replicated 3 times and relative gene expression was calculated by the delta-delta CT method.

## 3 Results

### 3.1 Induction of somatic embryogenesis

**Fig. 1:**
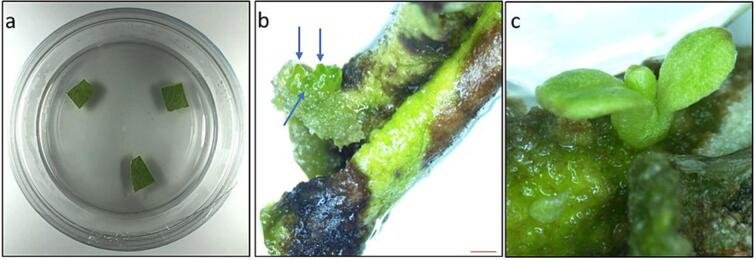
Leaf tissues of *A. africana* at the three different developmental stages during somatic embryogenesis. (a) Non embryonic leaf (NEL) at day 0 in culture medium. (b) Globular embryo formed leaf (GEL) after 21 days in culture medium. (c) Cotyledonary embryo formed leaf (CEL) after 28 days in culture medium. Bars: (b and c) = 1 mm.

After twenty-one days of culture, globular structures were formed on the *A. africana* leaf explants in MS medium augmented with BAP (1.0 mg/L), AMP (3.472 x 10^-2^ mg/L), and gelrite (3 g/L) (induction medium). After twenty-eight days from initial culture, leaf explants (transferred from induction medium at twenty-four days) developed cotyledonary embryos in MS medium fortified with ABA (0.5 mg/L), NAD (6.634 x 10^-2^ mg/L), and gelrite (9 g/L) (differentiation and maturation medium). NEL, GEL, and CEL served as samples for RNA extraction (Fig. 1**a, b, c**).

### 3.2 Sequencing, de novo assembly of transcriptome, and annotation

From the three sample groups, sequencing of the cDNA libraries generated 52,431,340 (Rep 1) and 53,156,748 (Rep 2) clean reads for NEL; 51,694,872 (Rep 1) and 53,823,646 (Rep 2) for GEL; and 53,902,776 (Rep 1) and 53,512,356 (Rep 2) for CEL (Table 1). Across all the sequence datasets, percentage of Q20 and Q30 were more than 95% and 90%, respectively (Table 1).

**Table 1:** Summary of transcriptome sequencing of leaf tissues from the three distinct somatic embryogenesis developmental stages (two replicates each) in *A. africana.*

**Sample**	**Sequence Length**	**Number of raw reads**	**Number of clean reads**	**Size of clean reads**	**GC%**	**Q20 (%)**	**Q30 (%)**
NEL-Rep 1	101	53,297,154	52,431,340	52.4 M	43.8283	97.745	93.8746
NEL-Rep 2	101	54,209,502	53,156,748	53.2 M	43.7386	97.6562	93.7953
GEL-Rep 1	101	52,372,398	51,694,872	51.7 M	45.6819	97.9179	94.1908
GEL-Rep 2	101	54,587,094	53,823,646	53.8 M	46.2483	97.9271	94.147
CEL-Rep 1	101	54,618,650	53,902,776	53.9 M	46.8022	97.946	94.1745
CEL-Rep 2	101	54,161,868	53,512,356	53.5 M	47.0983	98.031	94.3383

NEL (Non-embryonic leaf), GEL (Globular embryo formed leaf), CEL (Cotyledonary embryo formed leaf), Rep (Replicate).

**Fig. 2:**
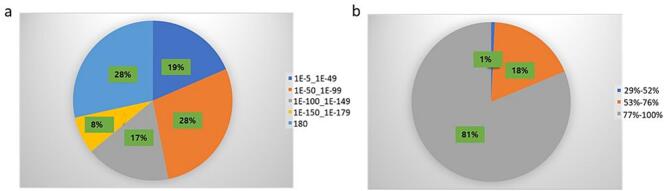
Homology features of *A. africana* Sequences in Nr Database (a) BLAST hit E-value distribution. (b) BLAST Hit similarity distribution of assembled Transcripts.

**Fig. 3:**
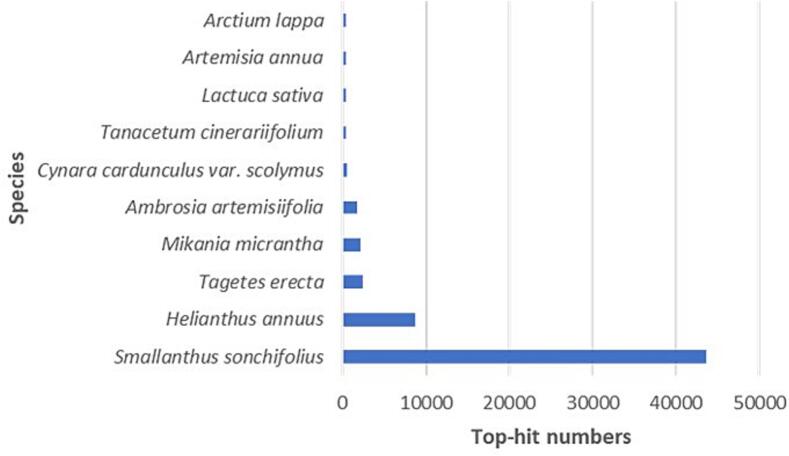
Number of transcripts aligning to top 10 Species via BLASTX.

A total of 318,521,738 clean reads generated from 323,246,666 raw reads (98.54%) were part of the assembly (Table 1). The trinity package generated 200,130 transcripts, averaging 822.11 bp in length (Table 2). Of the 200,130 transcripts, 27,345 (13.66%) and 62,930 (31.44%) were annotated by SwissProt and Nr database, respectively (Table 3). Top-hit E-value analysis showed 81% of sequences with marked Nr homology (<1E-50) and 81% with over 75% similarity (Fig. 2**a and b**). The three top-hit species were *Smallanthus sonchifolius*, *Helianthus annuus*, and *Tagetes erecta* with annotated transcripts of 43622, 8763, and 2406, respectively (Fig. 3). Accordingly, all the three plant species belong to the same family of Asteraceae with *A. africana*.

**Table 2:** Transcriptome assembly and predicted unigenes statistics.

**Type**	**Transcripts**	**Predicted Unigenes**
Total Sequences	200,130	64,481
Total Base	164,528,981	77,617,089
Average length (bp)	822.11	1,203.72
N50 length (bp)	1,259	1,503

**Table 3:** Annotation of assembled *A. africana* unigenes.

**Data base for annotation**	**Annotated unigenes count**	**Percentage (%)**
SwissProt	27,345	13.66
Nr	62,930	31.44

Regarding the function of somatic embryogenesis induction, 609 unigenes were recorded as putative homologs of genes associated with SE (Supplementary material 2). The most dominant groups included *Ethylene Responsive Factor* (*ERF*, 101 unigenes), *Heat-Shock proteins* (*HSPs*, 80 unigenes), *Leafy Cotyledon* (*LEC*, 70 unigenes), *APETALA2* (AP2, 67 unigenes), and *Auxin Responsive Factor* (*ARF*, 46 unigenes) (Supplementary material 2).

**Fig. 4:**
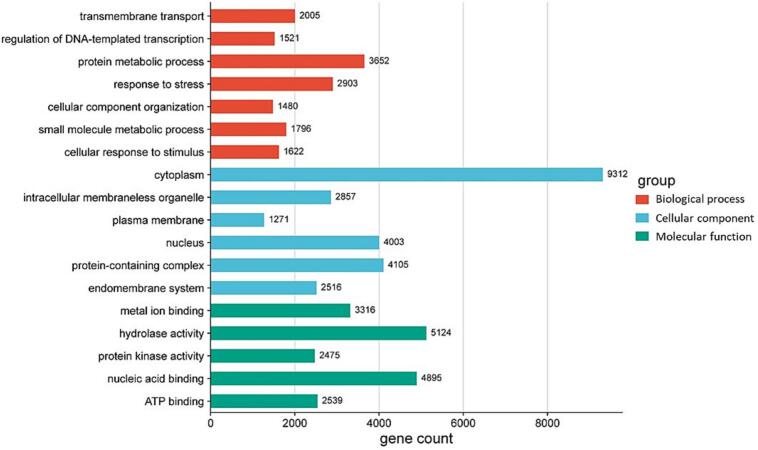
Gene ontology (GO) categorization of *A. africana* transcriptome by Blast2GO. Annotation of unigenes was performed across molecular function, cellular component, and biological process categories.

**Fig. 5:**
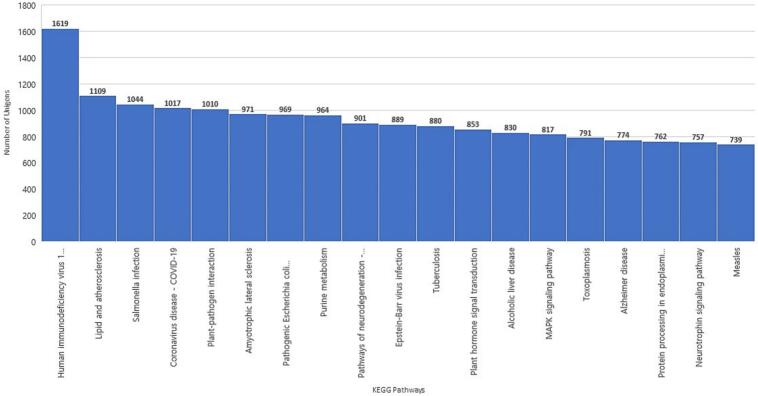
Unigene Classification in Kyoto Encyclopaedia of Genes and Genomes (KEGG) metabolic Pathways.

GO assignment classified the functions of the predicted unigenes into three GO-term categories of molecular function, cellular component, and biological process (Fig. 4). From each category, the top two dominant terms were “hydrolase activity” and “nucleic acid binding” (under molecular function), “cytoplasm” and “protein-containing complex” (under cellular component), as well as “protein metabolic process” and “response to stress” (under biological process) (Fig. 4). After KEGG pathways search, a total of 17,696 unigenes were assigned to 19 pathways (Fig. 5). Notably, several unigenes were classified into plant related pathways, for example, plant-pathogen interaction (1,010), purine metabolism (964), plant hormone signal transduction (853), and MAPK signaling pathway (817) (Fig. 5).

### 3.3 Differentially expressed genes

**Fig. 6:**
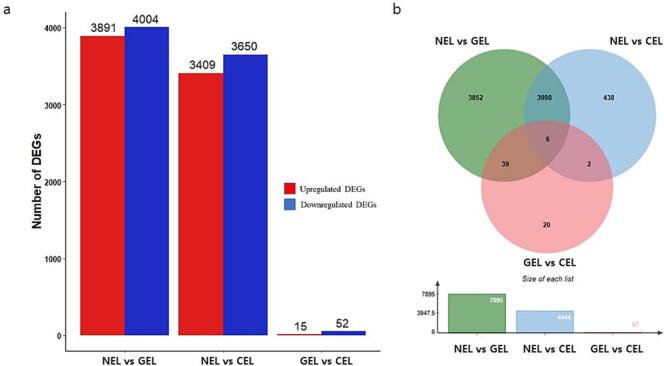
Histogram and Venn diagram of differentially expressed genes (DEGs) during somatic embryogenesis in *A. africana.* (a) Histogram of up- and down-regulated DEGs between different libraries. (b) Venn diagram showing DEGs in NEL vs GEL, NEL vs CEL, and GEL vs CEL. NEL = non embryonic leaf, GEL = globular embryo formed leaf, CEL = cotyledonary embryo formed leaf.

A total of 8669 DEGs were recorded when the 3 libraries were compared in pairs (Fig. 6**b and** Supplementary material 3). During SE, more genes exhibited down-regulation than up-regulation (Fig. 6**a**). Between each two libraries, NEL vs GEL (7,895), NEL vs CEL (7,059), and GEL vs CEL (67) unigenes were differentially expressed (Fig. 6**b**). Of the DEGs, 21 unigenes were differentially expressed across all the three comparisons. A total of 1,569 unigenes were differentially expressed only between NEL and GEL, 752 unigenes between NEL and CEL, and 17 unigenes between GEL and CEL (Fig. 6**b**). From the 3 libraries, the top 30 most expressed genes are provided in Supplementary material 4. These findings suggest that induction of SE using a combination of PGRs and other molecules (AMP and NAD) resulted into differential gene expression.

### 3.4 DEG functional annotation

**Fig. 7:**
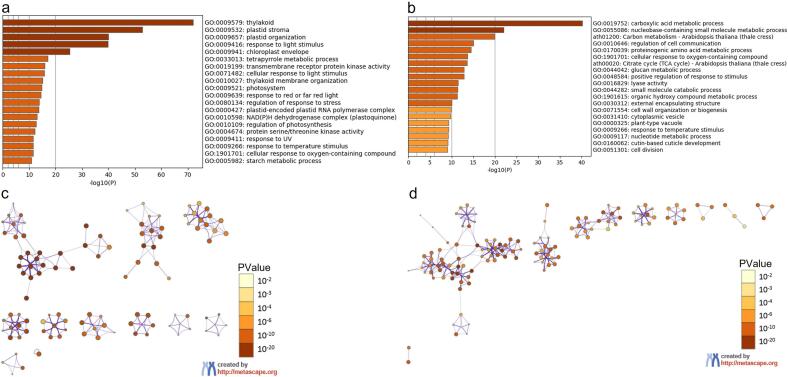
Metascape, GO and KEGG pathway functional enrichment analysis of DEGs during somatic embryogenesis in *A. africana.* (a) Significantly enriched GO terms and KEGG pathways of down-regulated genes. (b) Significantly enriched GO terms and KEGG pathways of up-regulated genes. (c) Network contact of GO terms and KEGG pathways of down-regulated genes. (d) Network contact of GO terms and KEGG pathways of up-regulated genes.

Functional enrichment analysis of DEGs showed results in four parts of molecular function, cellular component, biological process, and KEGG (Fig. 7**a, b, c, d**). The group of down-regulated genes, in molecular function, was mainly linked to activities such as transmembrane receptor protein kinase activity, protein serine/threonine kinase activity, transmembrane signaling receptor activity, and transmembrane receptor protein tyrosine kinase activity. In biological process, the genes were majorly concentrated in processes such as plastid organization, response to light stimulus, tetrapyrrole metabolic process, cellular response to light stimulus, thylakoid membrane organization, regulation of response to stress, and regulation of photosynthesis. In cellular component, the genes were enriched in thylakoid, plastid stroma, chloroplast envelope, photosystem, plastid-encoded plastid RNA polymerase complex, and NAD(P)H dehydrogenase complex (plastoquinone) (Fig. 7**a, c**).

On the other hand, the group of up-regulated genes exhibited a different network (Fig. 7**b, d**). In molecular function, the genes were majorly concentrated in activities like lyase activity, oxidoreductase activity, acting on NAD(P)H, primary active transmembrane transporter activity, vitamin binding, and transmembrane receptor protein kinase activity. In biological process, the genes were mainly concentrated in processes such as carboxylic acid metabolic process, nucleobase-containing small molecule metabolic process, regulation of cell communication, proteinogenic amino acid metabolic process, positive regulation of response to stimulus, small molecule catabolic process, and cell division. In cellular component, the genes were significantly enriched in cytoplasmic vesicle, plant-type vacuole, external encapsulating structure, cell wall, intracellular vesicle, oxidoreductase complex, organelle subcompartment, and nucleolus. In the KEGG pathway, genes were highly concentrated in carbon metabolism and citrate cycle (TCA cycle). This result implies that all important functions necessary for somatic cells to gain embryogenic competence are markedly enriched and over-represented.

### 3.5 Differential expression analysis of plant hormones signaling pathway related genes during A. africana SE

**Fig. 8:**
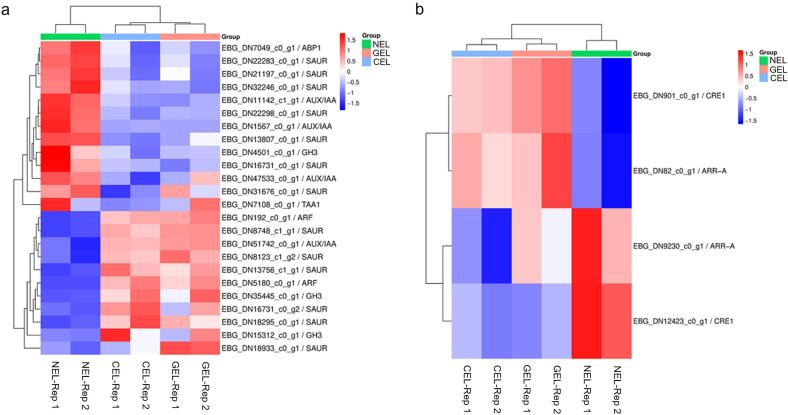
Heatmap of DEGs in auxin and cytokinin signalling pathway during somatic embryogenesis in *A. africana.* (a) Auxin signal transduction. (b) Cytokinin signal transduction. Heatmap show normalized data of RNA sequencing (Log2 CPM) with a rainbow color scale, each row corresponds to one gene, the IDs and names of DEGs are indicated to the right of the histograms, and each column corresponds to a sample. NEL = non embryonic leaf, GEL = globular embryo formed leaf, CEL = cotyledonary embryo formed leaf.

In this study, the representative pathways as per KEGG annotation were plant hormone signal transduction, phenylpropanoid biosynthesis, and tryptophan metabolism. Several genes involved in cytokinin (4 DEGs) and auxin (24 DEGs) biosynthesis, as well as signal transduction pathway were differentially expressed in tissues at different stages of embryogenesis (Supplementary material 5). For instance, genes involved in auxin signal transduction with markedly upregulated expression level (between non-embryogenic and embryogenic tissues) included, ARF (EBG_DN192_c0_g1 / ARF5 and EBG_DN5180_c0_g1 / ARF4), SAUR (EBG_DN18933_c0_g1 / SAUR, EBG_DN8748_c1_g1 / SAUR, EBG_DN18295_c0_g1 / SAUR, EBG_DN8123_c1_g2 / SAUR, EBG_DN16731_c0_g2 / SAUR, and EBG_DN13756_c1_g1 / SAUR), GH3 (EBG_DN35445_c0_g1 / GH3 and EBG_DN15312_c0_g1 / GH3), and AUX/IAA (EBG_DN51742_c0_g1 / AUX/IAA9) (Fig. 8**a**). On the other hand, genes involved in auxin signal transduction with significantly downregulated expression level included AUX/IAA (EBG_DN47533_c0_g1 / AUX/IAA, EBG_DN11142_c1_g1 / AUX/IAA, and EBG_DN1567_c0_g1 / AUX/IAA), SAUR (EBG_DN31676_c0_g1 / SAUR, EBG_DN22298_c0_g1 / SAUR, EBG_DN16731_c0_g1 / SAUR, EBG_DN13807_c0_g1 / SAUR, EBG_DN32246_c0_g1 / SAUR, EBG_DN21197_c0_g1 / SAUR, and EBG_DN22283_c0_g1 / SAUR), GH3 (EBG_DN4501_c0_g1 / GH3) and TAA1 (EBG_DN7108_c0_g1 / TAA1) (Fig. 8**a**). Genes involved in cytokinin signal transduction with upregulated expression level during SE in *A. africana* were CRE1 (EBG_DN901_c0_g1 / CRE1) and ARR-A (EBG_DN82_c0_g1 / ARR-A), while those downregulated were ARR-A (EBG_DN9230_c0_g1 / ARR-A) and CRE1 (EBG_DN12423_c0_g1 / CRE1) (Fig. 8**b**).

Additionally, many genes involved in ethylene, gibberellin, jasmonic acid, salicylic acid, abscisic acid (for example, PYL4), and brassinosteroid signal transduction pathway were differentially expressed during SE in *A. africana* (Supplementary material 5). This finding indicates that hormones and their crosstalk play a key role during SE in *A. africana*, thus plant hormone signaling pathways could serve as a primary regulator of the process.

### 3.6 Differentially expressed somatic embryogenesis candidate genes

Literature review was conducted using annotation and sequence similarity in order to identify main candidate genes reported previously in other species and present in this study. The candidate SE associated genes in *A. africana* are presented in (Table 4 and Supplementary material 2). Somatic embryogenesis receptor kinase (SERK) family is involved in embryogenic competence acquisition ^6^. In this work, a differentially expressed *A. africana* gene (EBG_DN12831_c0_g1) was identified with SwissProt entry linked to *Oryza sativa* SERK2. The SERK2 expression increased 3.9-fold during SE in *A. africana*.

**Table 4:** Somatic embryogenesis related genes reported in SwissProt and present in DEGs of *A. africana* during somatic embryogenesis.

**Gene name**	**SwissProt entry**	**Species**
SERK2	sp|Q7XV05|SERK2_ORYSJ	*Oryza sativa*
ERF12	sp|Q94ID6|ERF81_ARATH	*Arabidopsis thaliana*
ERF011	sp|Q9SNE1|ERF11_ARATH	*Arabidopsis thaliana*
ERF2	sp|Q40479|ERF2_TOBAC	*Nicotiana tabacum*
ARF5	sp|P93024|ARFE_ARATH	*Arabidopsis thaliana*
IAA9	sp|Q38827|IAA9_ARATH	*Arabidopsis thaliana*
PYL4	sp|O80920|PYL4_ARATH	*Arabidopsis thaliana*
LEA14-A	sp|P46518|LEA14_GOSHI	

Through the action of auxin response factor (ARF) transcription factors, auxin triggers downstream signaling, and previous assessment of SE efficiency in ARF mutants indicated that seven ARFs, especially ARF5, play roles in Arabidopsis SE induction ^32^. In *A. africana* SE, the expression of ARF5 (EBG_DN192_c0_g1) increased by 18.2-fold. Additionally, Auxin/Indole-3-Acetic Acids (Aux/IAAs) are reported to be auxin-responsive genes involved in auxin signaling and homeostasis, enabling precise and rapid regulation of downstream targets and influencing plant growth and development ^33^. In the present study, IAA9 (EBG_DN51742_c0_g1) expression increased by 2.5-fold. On the other hand, PYR/PYL/RCAR family functions as an ABA receptor within the ABA signaling pathway, and ABA signaling is reported to promote cell totipotency. During *A. africana* SE, the expression of PYL4 (EBG_DN22108_c0_g1) increased 2.9 times.

BABYBOOM (BBM) and other APETALA2 (AP2)/ ethylene-responsive element (ERF) family of transcription factors are useful in maintaining meristematic state of shoot and root meristems ^15^. During the process of SE in *A. africana*, ERF12 (EBG_DN40092_c0_g4), ERF11 (EBG_DN945_c1_g2), and ERF2 (EBG_DN2545_c0_g1) expression increased by 10.1-, 4.7-, and 6.1-fold, respectively.

**Fig. 9:**
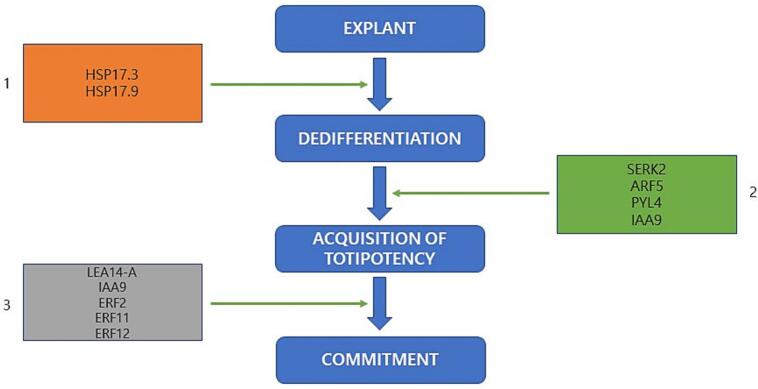
A proposed model showing different genes at different stages of SE pathway in *A. africana.* Rectangle 1 (coloured orange) shows genes involved in dedifferentiation. Dedifferentiation is the process in which a fully specialized cell returns to a less specialized state from within its own lineage, and this increases the developmental potency of cells. Rectangle 2 (coloured green) shows gene(s) involved in acquisition of totipotency by cells. Totipotency is the ability of a single plant cell to regenerate into a whole plant. Rectangle 3 (coloured gray) shows genes involved in commitment of totipotent cells to embryogenic state. HEAT SHOCK PROTEIN 17.3 and 17.9 (HSP17.3 and HSP17.9), SOMATIC EMBRYOGENESIS LIKE RECEPTOR KINASE 2 (SERK2), AUXIN RESPONSE FACTOR 5 (ARF5), PYRABACTIN RESISTANCE 1-LIKE 4 (PYLA4), AUXIN/INDOLE-3-ACETIC ACID GENE 9 (IAA9), LATE EMBRYO ABUNDANT 14-A (LEA14-A), ETHYLENE-RESPONSIVE TRANSCRIPTION FACTOR 2, 11, and 12 (ERF2, ERF11, and ERF12). (For interpretation of the references to colour in this figure legend, the reader is referred to the web version of this article.)

LATE EMBRYO ABUNDANT (LEA) proteins, which are highly expressed during later embryogenesis stages, influence the process of somatic and zygotic embryogenesis as well as stress associated responses ^15^. There expressions are almost exclusive in embryogenic tissues, other than in vegetative cells. In this study, transcript EBG_DN5644_c0_g1 annotated as LEA14-A increased in expression by 13.7-fold. Fig. 9 presents a possible model illustrating the transcriptional mechanisms involved during SE in *A. africana*.

### 3.7 Confirmation of somatic embryogenesis-related DEGs by qRT-PCR

**Fig. 10:**
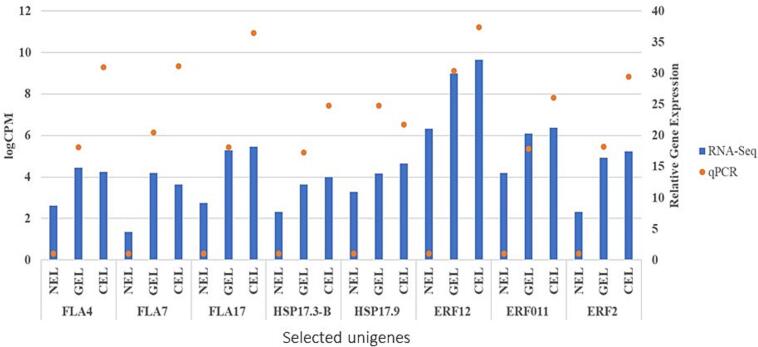
Expression levels of some somatic embryogenesis related genes in various tissues revealed by qPCR. The qPCR data are means of three replicates. NEL = non embryonic leaf, GEL = globular embryo formed leaf, CEL = cotyledonary embryo formed leaf.

**Fig. 11:**
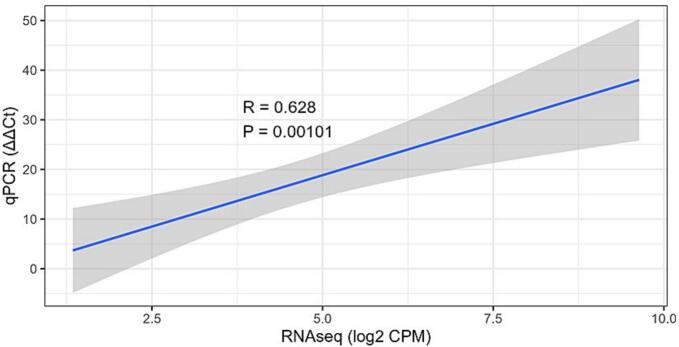
Correlation between qPCR and RNA-seq. Comparison of log2 fold change of 8 DEGs related to somatic embryogenesis obtained by qPCR and RNA-seq for NEL, GEL, and CEL. NEL = non embryonic leaf, GEL = globular embryo formed leaf, CEL = cotyledonary embryo formed leaf.

qRT-PCR analysis revealed that all the eight selected SE related unigenes were expressed at different levels during various stages of SE (Fig. 10). The unigenes, FLA4, FLA7, FLA17, HSP17, HSP17.3-B, ERF12, ERF011, and ERF2 were highly expressed in GEL and CEL tissues, while low in NEL tissues. These results confirmed that combination of plant hormones and molecules (AMP and NAD) led to differential expression of SE-associated genes, and these DEGs possibly play key roles during SE in *A. africana.* qRT-PCR verification of SE-related genes showed moderate correlation between RNA-seq and qRT-PCR data (R = 0.628), indicating the reliability of the RNA-seq analysis (Fig. 11).

## 4 Discussion

### 4.1 Induction of somatic embryogenesis

*A. africana* is an important medicinal plant and SE could facilitate its synthetic seed production, clonal plant regeneration, cryopreservation, and genetic improvement. Consequently, elucidating the molecular mechanisms that regulate embryogenesis is essential for the effective manipulation and optimization of SE. While SE-associated genes have been extensively characterized in *Arabidopsis thaliana*, no such genes have been reported to date in *A. africana*. This work presents a *de novo* assembled transcriptome along with extensive gene expression data during SE in *A. africana*, induced by a combination of plant hormones and other molecules (AMP and NAD).

As demonstrated in the Results, globular embryos developed on the surface of *A. africana* leaf explants in MS medium augmented with BAP (1.0 mg/L), AMP (3.472 x 10^-2^ mg/L), and gelrite (3 g/L). The embryos were matured in MS medium fortified with ABA (0.5 mg/L), NAD (6.634 x 10^-2^ mg/L), and gelrite (9 g/L) (Fig. 1**c and** Supplementary material 6). The significance of auxins and cytokinins in initiating an embryogenic response may lie in their role in regulating the cell cycle and promoting cell division ^34^. BAP efficacy in inducing SE observed in this study is probably due to its metabolic stability in *in vitro* cultures and its strong capacity to trigger cell division ^35^. Similarly, other researchers have reported optimal induction of SE in various plant species on media containing BAP, such as *Metabriggsia ovalifolia* W. T. Wang ^36^. In addition to BAP, the inclusion of AMP in the induction medium likely contributed to the successful formation of globular embryos observed at day 21. Exogenous metabolites such as AMP have been shown to enhance SE in various plant species ^9^. Adenosine, a key component of AMP is known to be a primary substrate for purine salvage, which influences growth and development of embryos ^37^. During the early stages of SE, purines are thought to be actively utilized to boost the internal nucleotide pool, which is essential for supporting the rapid cell proliferation and division of embryogenic tissues, in the presence of BAP and/or 2,4-D ^37^. Lee et al ^9^ reported that AMP promotes plant regeneration through a PLETHORA (PLT)-dependent mechanism; PLT genes (for example, PLT3 and PLT5) are central regulators of root primordium and meristem identity. Together, these findings indicate that AMP may be a key metabolite responsible for enabling embryogenic competence during SE in *A. africana*. AMP was also reported to improve SE in *Peucedanum japonicum* Thunb. ^9^.

Regarding maturation of embryos, the ability of ABA to promote embryo maturation may be due to its role in triggering vital metabolic changes that support reserve accumulation, leading to higher levels of fatty acids and storage proteins in somatic embryos ^38^. Effectiveness of ABA in improving maturation of somatic embryos has also been observed in other species including *Pinus elliottii* Engelm. ^39^. The capacity of NAD to enhance the maturation of *A. africana* somatic embryos could possibly be attributed to its role as a fundamental component of the NAD(P)+/NAD(P)H redox couple, which plays a an important role in cellular redox balance in plant cells ^40^. Redox control is crucial for regulating cell-cycle entry, progression, and exit, thereby affecting dedifferentiation, cell division, differentiation, and cell death ^41^. Accordingly, dedifferentiation is an early event in SE and is closely linked to chromatin remodeling ^40^, indicating that NAD may promote the induction of SE by triggering DNA hypomethylation, thereby reducing overall DNA methylation levels, as embryogenic tissues are typically characterized by lower DNA methylation than non-embryogenic tissues 41, 42. Moreover, an oxidizing cellular environment has been shown to support the completion of embryogenesis by enhancing processes such as shoot apical meristem (SAM) functionality ^43^, providing further mechanistic insight into the successful maturation of cotyledonary embryos observed in this study.

### 4.2 Sequencing, de novo assembly of transcriptome, and annotation

RNA-Seq data obtained from the three samples (NEL, GEL, and CEL) was used to assemble a *de novo* reference transcriptome in order to address the absence of reference for *A. africana* genome. As reflected in the Results, a total 200,130 transcripts with an average size of 822.11 bp were assembled from the pooled datasets. Following a similar approach, 186,203 and 205,592 assembled transcripts were identified during transcriptome analysis of *Garcinia mangostana* L. and *Cinnamomum camphora* L., respectively 6, 8. The differences registered in the number of transcripts assembled likely reveal the true variation in the loci being expressed across each sample and stages of development analysed ^6^. This suggests that the transcriptome assembled in this study is robust and has high-coverage.

In our work, 31.44% of the assembled transcripts were annotated by Nr database, implying that 68.56% of the sequences did not have clear homologs. This finding indicates that some of the genes may have novel functions. The small number of annotated transcripts may be because most sequences in the database were not obtained from samples collected during embryogenic development and limited number of sequences on phylogenetically closely related species in public databases ^6^. Furthermore, sequences lacking annotations might correspond to regions that are poorly conserved ^44^. Numerous studies have likewise reported a substantial number of unannotated transcripts 6, 45.

In this study, out of the 8,669 DEGs, the number of down-regulated genes were higher than up-regulated genes, triggering embryogenesis in somatic cells, and this is consistent with the other reports 6, 8. Carbon metabolism plays an important role in *A. africana* SE, as the majority of DEGs were mapped to carbon metabolism pathway. Compared to other carbohydrates, sucrose has commonly been regarded as the most effective carbon and energy source supporting plant tissue growth and development. It has been revealed that sucrose serves as a primary transport sugar and sometimes regulates gene expression ^6^.

### 4.3 Differential expression analysis of plant hormones signaling pathway related genes during a. Africana SE

The KEGG pathway enrichment analysis presented in the Results identified plant hormone signal transduction, phenylpropanoid biosynthesis, and tryptophan metabolism as the most representative pathways during SE in *A. africana*. This enrichment highlights the central role of phytohormone-mediated signaling and metabolic reprogramming in regulating the transition from non-embryogenic to embryogenic tissues. Plant hormones are signaling compounds generated within the plant in very small amounts and they regulate several processes such as determining the formation of leaves, flowers, embryogenesis, fruit ripening and development, and response to abiotic and biotic stress ^46^. Through a series of signal transduction components, plant hormones are perceived and transmitted to the nucleus to regulate expression of genes, leading to several physiological processes ^6^. In growth media, the concentrations of exogenous hormones affect that of endogenous hormones, and zeatin and IAA are the key endogenous hormones that influence embryogenesis 6, 34. In higher plants, tryptophan is considered to be the most important IAA biosynthesis pathway, and its metabolism has been reported when embryogenesis occurs ^6^. In the present study, some genes involved in tryptophan metabolisms and zeatin biosynthesis showed increased expression (Supplementary material 5), suggesting their involvement in SE of *A. africana*. However, genes that encode auxin-responsive protein IAA (AUX/IAA), for example EBG_DN47533_c0_g1 / AUX/IAA and some Small Auxin-up RNAs (SAUR) family proteins, were down-regulated. Influence of zeatin biosynthesis and tryptophan metabolism on embryogenesis is dependent on transportation, signal transduction, as well as degradation ^6^. Notably, the up-regulation of genes associated with zeatin signal transduction, such as EBG_DN901_c0_g1 / CRE1, which encode cytokinin receptors, implies that cytokinin plays a key role in SE of *A. africana*. Accordingly, transcripts associated with auxin were more than those associated with cytokinin during SE in *A. africana*. This finding is in agreement with that of Xu et al ^47^ who also recorded higher number of transcripts related to auxin than those related to cytokinin during SE in cotton.

### 4.4 Differentially expressed somatic embryogenesis candidate genes

During SE, genes have been selectively expressed and it is valuable to identify and characterize them 6, 8. In the current study, many SE-related genes were differentially expressed during SE. Upregulated SE-related genes included those from the family of SERK, ERF, LEA, GH3, HSP, and ARF. Auxins have been the most widely used to induce SE in various plant species during *in vitro* culture 15, 34. During the auxin induced SE, many genes involved were isolated, including Aux/IAAs, SAURs, ARFs, GH3s, PINs, and HSPs ^15^. Accordingly, HSP family genes were reported to be upregulated during initiation of SE ^15^. In the present study, treating explants (leaf) with cytokinin (BAP), ABA, and molecules (AMP and NAD) resulted into differential expression of these SE related genes. This suggests that auxin-associated genes (especially ARF5 and IAA9) may still regulate induction of SE in *A. africana*, even without exogenous application of auxins. Mahdavi-Darvari & Noor ^6^ had similar findings although SE was induced using cytokinin (BAP and TDZ) in *Garcinia mangostana* L.

SERK is known to be the most relevant gene that increases its expression during induction of SE; in this study, the SERK2 gene (EBG_DN12831_c0_g1) exhibited a 3.9-fold increase in expression during SE. The SERK family of genes, which encodes proteins, is responsible for several developmental processes such as differentiation/transdifferentiation and totipotency, and it has been identified in embryogenic cells of various plant species such as *Daucus carota* and *Glycine max* 15, 16. SERK (a cell surface receptor) binds to the ligand via the leucine-rich repeat (LRR) domain and initiates a signal cascade, that with support of intracellular domains reaches the nucleus ^15^. Through chromatin remodeling, this cascade changes the pattern of gene expression ^48^. Usually, genes activity is changed via repressing selective or specific genes and alteration of expression of others ^15^. Sharp upregulation of SERK has been registered from embryogenic induction to globular stage, facilitating transition of non-embryogenic tissues to embryogenic cells, in combination with other genes ^49^. This implies that the SERK2 (EBG_DN12831_c0_g1) gene upregulated during SE of *A. africana* possibly plays an important role in acquisition of embryogenic competence. Like in this study, the expression of SERK2 in *Oryza sativa* increased to about three-fold in embryogenic callus and maturation phase, relative to non-embryogenic callus ^50^.

ABA signaling also emerged as a key regulatory pathway during SE, as evidenced in the Results by the 2.9-fold upregulation of PYL4 (EBG_DN22108_c0_g1). PYL proteins function as ABA receptors and are essential for ABA-mediated enhancement of SE efficiency ^51^. The increased expression of PYL4 gene indicates that it is one of the key drivers of ABA signaling, which in turn promotes cell totipotency ^51^. Relatedly, Chen et al ^52^ also reported that PYL4/9 were among the major transcriptome factors that influence SE induction in *Lilium Oriental × Trumpet* hybrid, reinforcing the relevance of ABA signaling in *A. africana* SE.

APETALA 2/ETHYLENE RESPONSE FACTOR (AP2/ERF) are DNA-binding type transcription factors that are encoded by BBM, and has been identified in embryogenic tissues of many plant species including *Brassica napus* ^15^. BBM together with AP2/ERF transcription factors facilitate shoot and root meristems to maintain meristematic state ^15^. AP2/ERF has the ability to bind to GCC box, a DNA sequence linked to ethylene response, and the family has also been heavily connected to induction of SE 15, 53. In this study, the increased expression of ERF12 (EBG_DN40092_c0_g4), ERF11 (EBG_DN945_c1_g2), and ERF2 (EBG_DN2545_c0_g1) suggest their possible role in maintaining the meristematic state of root and shoot meristems of *A. africana* embryos.

LEA is highly expressed at later stages of embryogenesis and its presence has been reported in several higher plants including *Arabidopsis thaliana* ^54^, *Oryza sativa* ^55^, and *Solanum lycopersicum* ^56^. LEA genes are regarded to play an important role in plant growth and development by minimizing effects of different stress conditions in cells ^57^. Noteworthy is that the LEA genes accumulate at final stages of seed maturation, coinciding with the start of dehydration, indicating their potential involvement in protecting the maturing seeds from environmental stress ^57^. The mechanisms through which LEA genes exert their protective effects against abiotic stress include maintaining membrane structural integrity and sequestering ions ^57^. In the present study, the highly expressed transcript LEA14-A (EBG_DN5644_c0_g1) may possibly be involved in *A. africana* embryo maturation and desiccation tolerance.

In this research, the minimal variation in gene expression observed in samples at globular and cotyledonary embryogenic stage (CEL vs. GEL) may be attributed to the possibly that in some species, at the globular stage, the key regulatory and transcriptional programs necessary for somatic embryo identity are already largely in place 16, 58. As development proceeds to the cotyledonary stage, additional changes are more about fine-tuning (for example, metabolism and maturation) rather than wholesale changes in transcriptional identity ^58^. Comparable to our finding, some other researchers also reported limited DEGs between somatic embryogenic tissues, as opposed to large number of DEGs recorded between non embryogenic and embryogenic tissues 59, 60.

## 5 Conclusion

In this study, analysis of *de novo* assembled transcriptomes of *A. africana* embryogenic tissues (NEL, GEL, and CEL) generated a substantial volume of sequence data. Profiles of gene expression were evaluated during SE induced by combining plant hormones with AMP and NAD. Differential expression of genes potentially facilitated acquisition of embryogenic competence and formation of somatic embryos. Differential expression of SE related genes suggests that mechanisms of SE induced by a combination of plant hormones with other molecules such as AMP and NAD may be the same or similar to that of SE induced by plant hormones alone. For the first time, this study provides insights into SE induced by a combination of plant hormones with other molecules, and offers beneficial resources for future genomic, transcriptomic, and genetic research on *A. africana*. Moreover, this work may provide an important basis for future functional research on plant embryogenesis.

## Data availability

The Illumina sequence data from this study have been submitted to the NCBI sequence read archives under the accession number [PRJNA1334589]. All the supporting data are included as supplementary materials.

## CRediT authorship contribution statement

**Roggers Gang:** Writing – original draft, Methodology, Investigation, Formal analysis, Conceptualization. **Ariranur Haniffadli:** Writing – review & editing, Methodology, Investigation, Formal analysis. **Daeui Park:** Writing – review & editing, Validation, Software, Formal analysis. **Chunggeon Lee:** Writing – review & editing, Validation, Software, Formal analysis. **Subramanian Muthamil:** Writing – review & editing, Validation, Software, Formal analysis. **Jun Hong Park:** Writing – review & editing, Validation. **Yeongjun Ban:** Writing – review & editing. **Youngmin Kang:** Writing – review & editing, Supervision, Project administration.

## Funding

This research was funded by the Development of Sustainable Application for Standard Herbal Resources (KSN1823320), Development of Innovative Technologies for the Future Value of Herbal Medicine Resources (KSN2511030) Korea Institute of Oriental Medicine, through the Ministry of Science and ICT, Republic of Korea.

## Declaration of competing interest

The authors declare that they have no known competing financial interests or personal relationships that could have appeared to influence the work reported in this paper.
