# Supplementary material for: De novo transcriptome analysis of somatic embryogenesis induced by plant hormones combined with adenosine 5-monophosphate and nicotinamide adenine dinucleotide molecules in Aspilia africana (Pers.) C.D.Adams
Source: J Genet Eng Biotechnol. 2026 Mar 27;24(2):100687. doi: 10.1016/j.jgeb.2026.100687 (PMC13054418; doi:10.1016/j.jgeb.2026.100687)
Supplement: Supplementary Data 3 [file mmc3.docx]

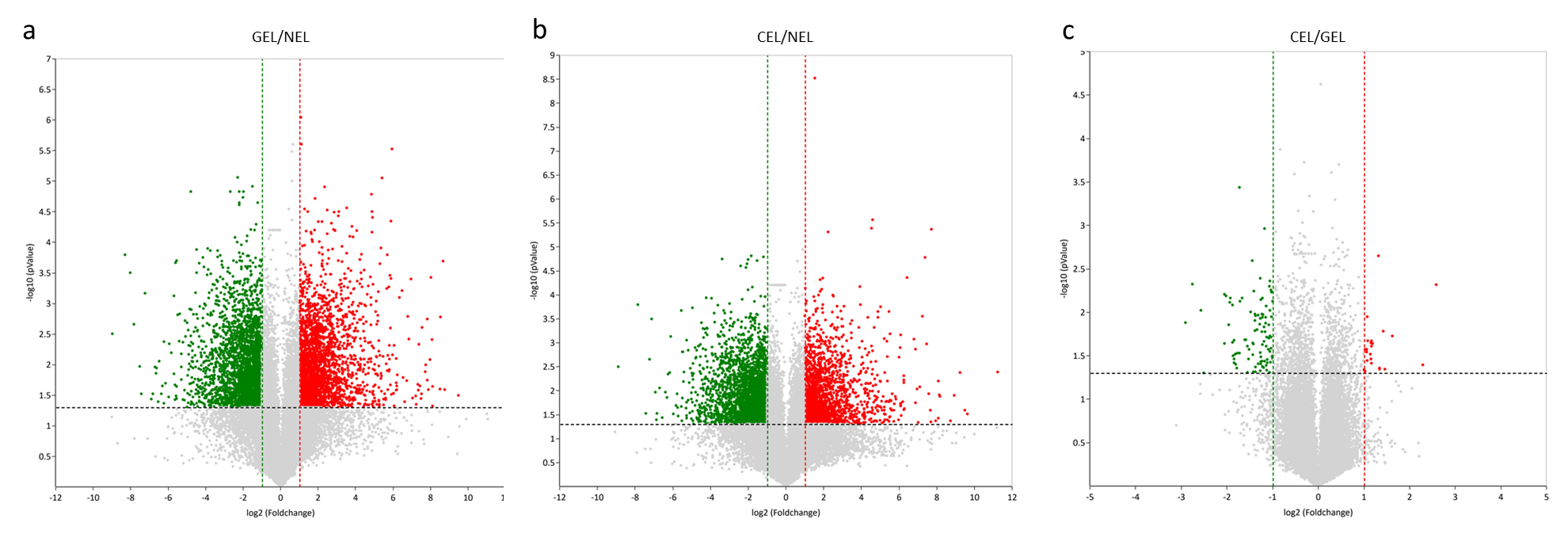


**Supplementary material 3.** Volcano plot of differential gene expression in non embryonic and embryonic leaf tissues. (a) GEL vs NEL. (b) CEL vs NEL. (c) CEL vs GEL. The fold change (group 1/group 2) of DEGs was relative to their expression in the three comparisons. DEGs with a positive fold change (red) were up-regulated in group 1 (down-regulated in group 2), and DEGs with a negative fold change (green) were down-regulated in group 1 (up-regulated in group 2). DEGs that were statistically significant (q-value<0.005, log_2_fold change >1) are shown in green and red, while those not statistically significant are shown in grey. NEL (Non-embryonic leaf), GEL (Globular embryo formed leaf), CEL (Cotyledonary embryo formed leaf).


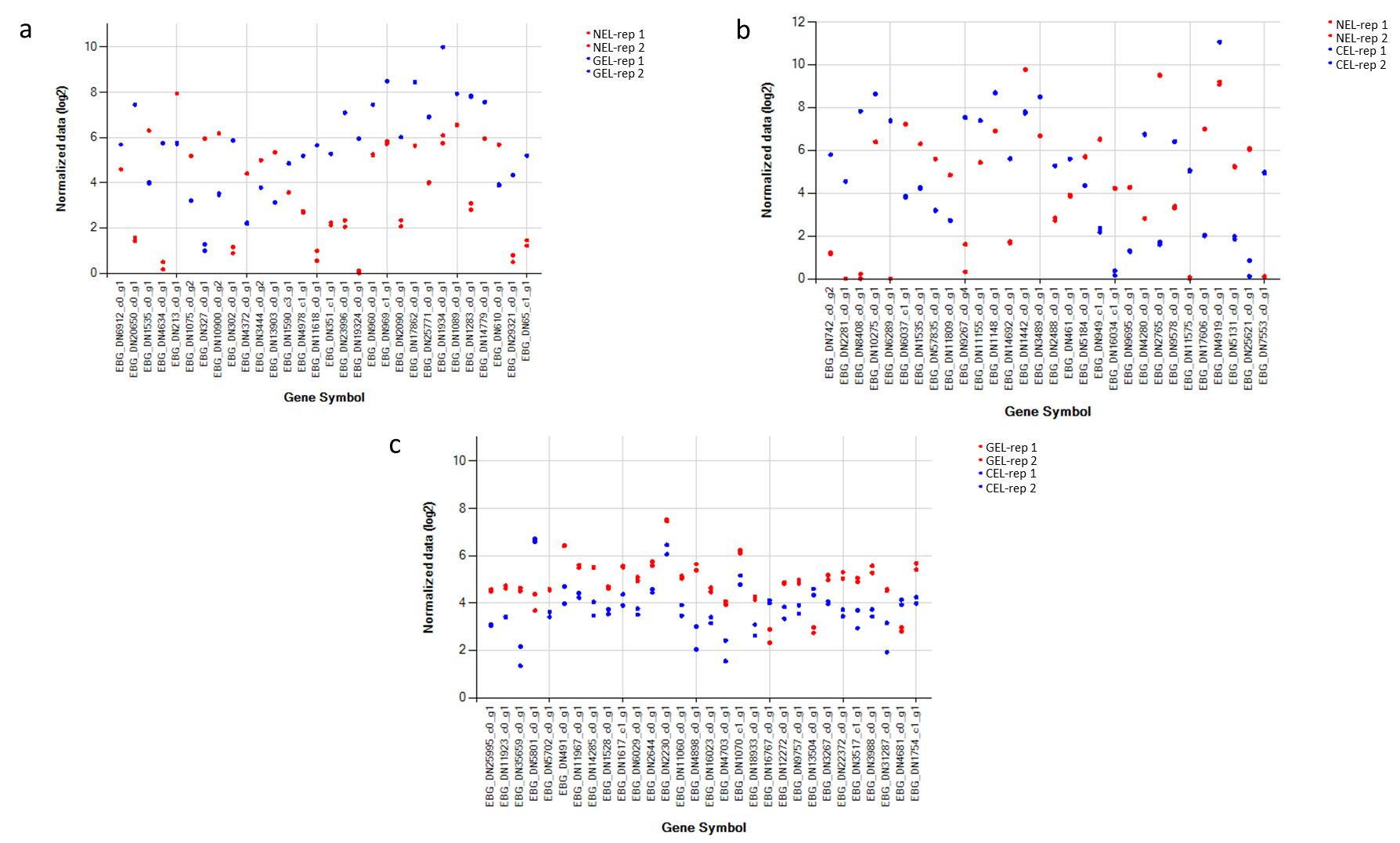


**Supplementary material 4.** The top 30 most expressed genes from (a) NEL and GEL libraries (b) NEL and CEL libraries (c) GEL and CEL libraries. NEL (Non-embryogenic leaf), GEL (Globular embryo formed leaf), CEL (Cotyledonary embryo formed leaf).


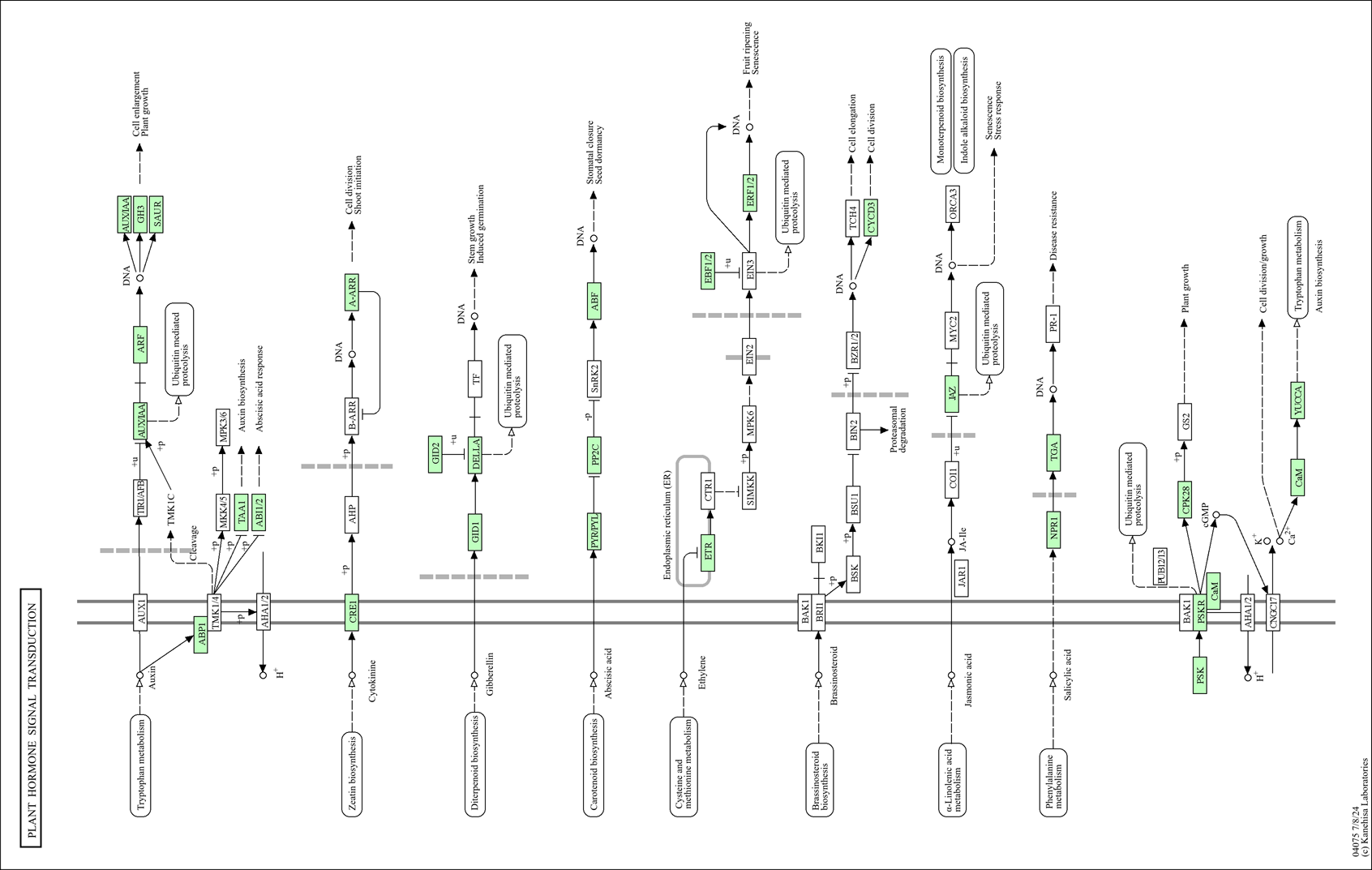


**Supplementary material 5.** Plant hormone signal transduction pathway during somatic embryogenesis in *A. africana.* Green frames represent differentially expressed transcript.


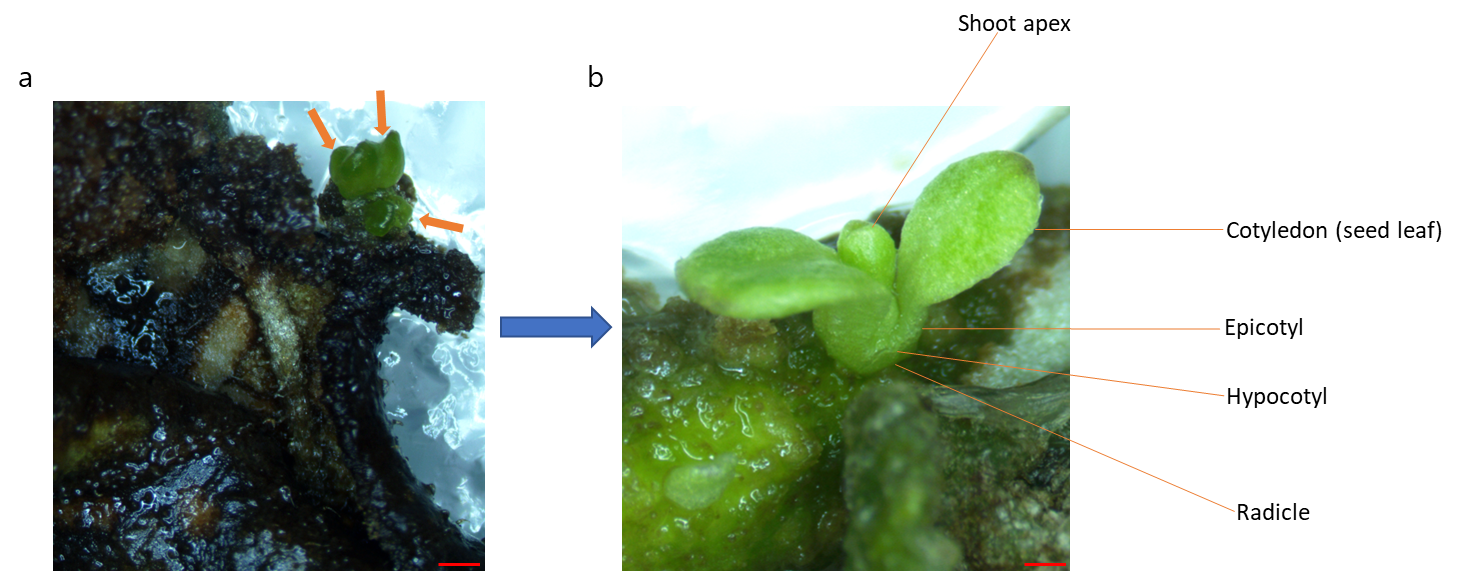


**Supplementary material 6.** Images of somatic embryos of *A. africana* formed from leaf explants. (a) Developing heart stage embryos (b) Cotyledonary somatic embryo with labelled key structures. Bars: (a and b) = 1 mm.
